# Lack of Significant Effects of *Chlamydia trachomatis* Infection on Cervical Adenocarcinoma Risk: Nested Case-Control Study

**DOI:** 10.1371/journal.pone.0156215

**Published:** 2016-05-26

**Authors:** Vitaly Smelov, Tarik Gheit, Karin Sundström, Alexander Ploner, Sandrine McKay-Chopin, Carina Eklund, Massimo Tommasino, Joakim Dillner

**Affiliations:** 1 Infections and Cancer Biology Group, International Agency for Research on Cancer, World Health Organization, Lyon, France; 2 Screening Group, International Agency for Research on Cancer, World Health Organization, Lyon, France; 3 Department of Laboratory Medicine, Karolinska Institutet, Stockholm, Sweden; 4 Department of Medical Epidemiology and Biostatistics, Karolinska Institutet, Stockholm, Sweden; Istituto Nazionale Tumori, ITALY

## Abstract

**Background:**

A role of *Chlamydia trachomatis* in HPV-induced cervical carcinogenesis has been reported for cervical cancer but studies on cervical adenocarcinoma are limited.

**Methods:**

A total of 1,553 cervical smears taken up to 26 years before diagnosis in a large population-based nested case-control study of cervical adenocarcinoma (AC, 132 cases with matched controls), and adenocarcinoma *in situ* (AIS, 159 cases with matched controls) were tested for *C*. *trachomatis* and HPV DNA by a type-specific PCR bead-based multiplex genotyping (TS-MPG) assay.

**Results:**

Only 1.7% of samples were positive for *C*. *trachomatis*, with no significant differences between AC/AIS cases and controls. HPV-positivity was detected in 49.3% of *C*. *trachomatis-negative* and 65.4% *C*. *trachomatis*-positive samples, respectively.

**Conclusions:**

A large prospective study did not find any risk for cervical adenocarcinoma and/or AIS conferred by *C*. *trachomatis* infection.

**Impact:**

*C*. *trachomatis* appears not to be involved in cervical adenocarcinomas.

## Introduction

Persistent infection with human papillomavirus (HPV) is a virtually necessary cause of cervical cancer [1]. One of the most common curable sexually transmitted infections (STI) worldwide *Chlamydia trachomatis* has been associated with an increased risk of cervical cancer [2–4]. While the most of the studies are focused on squamous cervical carcinoma, there was more limited power to study cervical adenocarcinoma, but no association was found [3,5], which is surprising as *C*. *trachomatis* primarily infects glandular cells [2]. Some studies relied on seroepidemiology, where it is difficult to rule out residual confounding completely [1]. One study reported no detection of *C*. *trachomatis* DNA in 71 archived formalin-fixed paraffin-embedded tissues of cervical adenocarcinoma (5), but cross-sectional studies are less informative than longitudinal studies [1] and *C*. *trachomatis* has only been detected in samples taken many years before the cancer, not close to the diagnosis [2].

We previously reported a large prospective study of cervical adenocarcinoma that established a strong association with HPV present many years before cancer diagnosis (6). We now wished to use this large prospective study to investigate possible associations between *C*. *trachomatis* and cervical adenocarcinoma.

## Materials and Methods

Detailed characteristics of the study have been reported previously [6]. A total of 1,553 β-globin-positive cervical smears collected during the pre-HPV vaccine era (1969–2002) in Sweden before the development of adenocarcinoma *in-situ* (AIS, 133 cases; mean age at entry and diagnosis: 28 and 37 years, respectively) or invasive cervical adenocarcinoma (AC, 170 cases; 37/43 years) and matched controls (128 and 169; 29/37 and 37/43 years, respectively) were tested for the presence of *C*. *trachomatis* and 19 mucosal HPV types (HPV-6, 11, 16, 18, 31, 33, 35, 39, 45, 51, 52, 56, 58, 59, 66, 68a and 68b, 70, 73 and 82), using a validated type-specific PCR bead-based multiplex genotyping (TS-MPG, IARC, Lyon, France) assay that combines multiplex polymerase chain reaction (PCR) and bead-based Luminex technology (Luminex Corp., Austin, TX, USA), as described elsewhere [7,8].

## Results

In total, 1.7% (26/1553) of samples were positive for *C*. *trachomatis* (Table 1). The 26 samples corresponded to only 21 distinct subjects. No major difference in *C*. *trachomatis*-positivity was observed between the outcomes and case-control status; the odds-ratios (ORs) for the association between chlamydia infection in the first collected smear and subsequent adenocarcinoma were 1.25 for AIS (95% CI 0.34–4.65) and 3.0 for AC (95%CI 0.31–28.84); ORs associated with chlamydia infection in the last smear before diagnosis were not estimable due to the lack of exposed cases (AIS) and controls (AC), respectively (Table 2). The full data set is available as Supplementary Information (S1 Table).

**Table 1:** Study population and distribution of *C*. *trachomatis* positive smears between cases and controls, for all β-globin-positive smears collected in the study.

	Adenocarcinoma *in situ* (AIS)		Invasive adenocarcinoma (AC)	
	Cases	Controls	Cases	Controls
Subjects	133	128	170	169
Age at first smear^a^	28 (17–71)	29 (16–71)	37 (17–82)	37 (17–82)
Age at last smear^a^	33 (17–72)	34 (17–71)	41 (20–82)	39 (20–82)
Age at diagnosis^a^	37 (20–75)	37 (20–75)	43 (25–88)	43 (25–89)
Time in study^a^	7.4 (0.1–27.0)	8.0 (0.2–25.9)	6.1 (0.0–21.1)	6.0 (0.1–22.2)
Smears^b^	425 (100)	332 (100)	422 (100)	374 (100)
HPV positive^b^	323 (76)	93 (28)	267 (63)	87 (23)
CT positive^b^	10 (2.4)	7 (2.1)	7 (1.7)	2 (0.5)

^a^ Reported in years: median (minimum-maximum)

^b^ Reported as count (percentage)

CT–*C*. *trachomatis* HPV–Human papillomavirus (any strain)

**Table 2:** Risk of cervical adenocarcinoma given by *C*. *trachomatis* infection in the first and last smear, calculated as odds ratio via conditional logistic regression where possible.

	Smear	Matched cases/controls	Exposed cases/controls^a^	Odds ratio	95% Conf.int.
AIS	First	128	5/4	1.25	0.34–4.65
	Last	128	0/2	NA^b^	NA^b^
AC	First	158	3/1	3.00	0.31–28.84
	Last	158	2/0	NA^b^	NA^b^

^a^ Matched case/control pairs that differ in exposure and contribute to the OR estimation, reported as exposed cases/exposed controls

^b^ Could not be calculated due to perfect separation (no exposed cases or controls)

Footnote: AIS–adenocarcinoma *in situ*, AC–adenocarcinoma

HPV-positivity was detected in 49.3% (n = 753) of *C*. *trachomatis*-negative and 65.4% (n = 17) *C*. *trachomatis*-positive samples. Because of the overall low numbers, a further analysis of possible interaction between *C*. *trachomatis* and HPV status was not feasible.

## Discussion

In the current largest prospective study to date of *C*. *trachomatis* and cervical adenocarcinoma, with a follow-up period of up to 26 years, *C*. *trachomatis* was not associated with increased risks of subsequent invasive adenocarcinoma (AC) and its precursor, adenocarcinoma *in situ* (AIS). The average age of participants in this study was above 30 years, and it is possible that co-infections/interactions between *C*. *trachomatis* and HPV, or other STI, may be of more relevance at earlier age and this may require further studying. A higher prevalence of HPV infections have been found in younger Italian women affected with a *C*. *trachomatis* chronic infection from a STI centre than in the ones from an assisted reproductive technology clinic [9]. HPV genotype distribution showed that mostly uncommon low risk genotypes were associated with *C*. *trachomatis* [10]. However, while Danish women who reported more than one Chlamydia infection had a statistically significantly increased risk of CIN3+, no association was found between *C*. *trachomatis* DNA and subsequent risk of CIN3+ among the ones who were HPV-positive or had a persistent HPV infection at baseline [11]. No association between *C*. *trachomatis* status, as assessed by DNA or IgG, and risk of cervical premalignancy, after controlling for carcinogenic HPV-positive status was found in a previous study from the United States [12], which suggested that positive associations between *C*. *trachomatis* and cervical premalignancy could have been caused, in part, by an increased susceptibility to HPV infection [12]. However, no studies have been done among the women with cervical adenocarcinoma and the present study is the first of this kind. Moreover, the reports of an interaction between *C*. *trachomatis* and HPV in squamous cell carcinoma of the cervix are mechanistically unexplained [1] and further studies on the co-factor role of genital microbiota in promoting malignancies, in particular in high-risk and younger populations, may be warranted.

## Supporting Information

S1 Table The analysis dataset on the detection of *Chlamydia trachomatis* and human papillomavirus (HPV) infections in women with cervical adenocarcinoma. SubjectID–unique subject identifier, RisksetID–unique risk set identifier, CaseStatus–case-control status (1 = case), AgeAtDiag–age at the time of diagnosis, HPVpos–HPV-positivity (1 = HPV-positive), CTpos–Chlamydia trachomatis-positivity (1 = Chlamydia-positive), AgeAtSmear–age at the time of collecting cervial smear).
(ODS)

## References

[1] International Agency for Research on Cancer. Monographs on the evaluation of carcinogenic risks to humans, volume 100 B, biological agents Lyon: IARC; 2012. 475 p.

[2] Wallin K-L, Wiklund F, Luostarinen T, Angström T, Anttila T, Bergman F, et al A population-based prospective study of Chlamydia trachomatis infection and cervical carcinoma. Int J Cancer. 2002 10 1;101(4):371–4. 12209962 10.1002/ijc.10639

[3] Smith JS, Bosetti C, Muñoz N, Herrero R, Bosch FX, Eluf-Neto J, et al Chlamydia trachomatis and invasive cervical cancer: a pooled analysis of the IARC multicentric case-control study. Int J Cancer. 2004 9 1;111(3):431–9. 15221973 10.1002/ijc.20257

[4] Arnheim Dahlström L, Andersson K, Luostarinen T, Thoresen S, Ögmundsdottír H, Tryggvadottír L, et al Prospective seroepidemiologic study of human papillomavirus and other risk factors in cervical cancer. Cancer Epidemiol Biomark Prev. 2011 12;20(12):2541–50. 10.1158/1055-9965.EPI-11-0761 21994401

[5] Quint KD, de Koning MNC, Geraets DT, Quint WGV, Pirog EC. Comprehensive analysis of Human Papillomavirus and Chlamydia trachomatis in in-situ and invasive cervical adenocarcinoma. Gynecol Oncol. 2009 9;114(3):390–4. 10.1016/j.ygyno.2009.05.013 19500822

[6] Dahlström LA, Ylitalo N, Sundström K, Palmgren J, Ploner A, Eloranta S, et al Prospective study of human papillomavirus and risk of cervical adenocarcinoma. Int J Cancer. 2010 10 15;127(8):1923–30. 10.1002/ijc.25408 20473898 PMC2930102

[7] Schmitt M, Dondog B, Waterboer T, Pawlita M, Tommasino M, Gheit T. Abundance of multiple high-risk human papillomavirus (HPV) infections found in cervical cells analyzed by use of an ultrasensitive HPV genotyping assay. J Clin Microbiol. 2010 1;48(1):143–9. 10.1128/JCM.00991-09 19864475 PMC2812266

[8] Bellaminutti S, Seraceni S, De Seta F, Gheit T, Tommasino M, Comar M. HPV and Chlamydia trachomatis co-detection in young asymptomatic women from high incidence area for cervical cancer. J Med Virol. 2014 11;86(11):1920–5. 10.1002/jmv.24041 25132162

[9] Seraceni S, De Seta F, Colli C, Del Savio R, Pesel G, Zanin V, et al High prevalence of hpv multiple genotypes in women with persistent chlamydia trachomatis infection. Infect Agent Cancer. 2014;9:30 10.1186/1750-9378-9-30 25621003 PMC4304071

[10] Seraceni S, Campisciano G, Contini C, Comar M. HPV genotypes distribution in Chlamydia trachomatis co-infection in a large cohort of women from North-East Italy. J Med Microbiol. 2016 3 3; 10.1099/jmm.0.000245 26944507

[11] Jensen KE, Thomsen LT, Schmiedel S, Frederiksen K, Norrild B, van den Brule A, et al Chlamydia trachomatis and risk of cervical intraepithelial neoplasia grade 3 or worse in women with persistent human papillomavirus infection: a cohort study. Sex Transm Infect. 2014 11;90(7):550–5. 10.1136/sextrans-2013-051431 24728044

[12] Safaeian M, Quint K, Schiffman M, Rodriguez AC, Wacholder S, Herrero R, et al Chlamydia trachomatis and risk of prevalent and incident cervical premalignancy in a population-based cohort. J Natl Cancer Inst. 2010 12 1;102(23):1794–804. 10.1093/jnci/djq436 21098758 PMC2994864

